# Mitotically active cellular fibroma of the ovary: a case report and literature review

**DOI:** 10.1186/s13048-015-0191-x

**Published:** 2015-10-06

**Authors:** Takashi Yamada, Kimiaki Hattori, Hidetoshi Satomi, Yoshinobu Hirose, Go Nakai, Atsushi Daimon, Atsushi Hayashi, Yoshito Terai, Masahide Ohmichi, Masaharu Fukunaga

**Affiliations:** Department of Pathology, Osaka Medical College, 2-7 Daigaku-machi, Takatsuki, Osaka 569-8686 Japan; Department of Radiology, Osaka Medical College, 2-7 Daigaku-machi, Takatsuki, Osaka 569-8686 Japan; Department of Obstetrics and Gynecology, Osaka Medical College, 2-7 Daigaku-machi, Takatsuki, Osaka 569-8686 Japan; Department of Pathology, Jikei Daisan Hospital, 4-11-1, Izumihoncho, Komaeshi, Tokyo 201-8601 Japan

**Keywords:** Mitotically active cellular fibroma, Ovary, Laparoscopy, Fibrosarcoma

## Abstract

**Background:**

The ovarian cellular fibrous tumor with mitotic figure >4 per 10 high power field without moderate to severe atypia is defined as mitotically active cellular fibroma according to the 2014 World Health Organization classification. As this category is new and rare now, we described here a case of MACF and reviewed the literature.

**Case:**

We present a case of mitotically active cellular fibroma of the ovary with 10-year history that was treated with laparoscopic surgery.

**Methods:**

We reviewed the relevant literature using PubMed search system and analyzed the previous cases.

**Results:**

To date, only 5 cases of mitotically active cellular fibroma have been reported. Our patient is the first case of mitotically active cellular fibroma of the ovary treated with laparoscopic surgery.

**Conclusion:**

MACF of the ovary is a newly defined category and few cases have been reported, while prognostic factors have also not yet been fully characterized. Long-term clinical follow-up is necessary.

## Background

The ovarian cellular fibrous tumors with mitotic figures >4 per 10 high-power fields (HPF) had been defined as fibrosarcoma [1]. In 2014 World Health Organization (WHO) histological classification, mitotic activity of >4 per 10 HPF in an ovarian cellular fibromatous neoplasm in the absence of moderate to severe atypia is defined as mitotically active cellular fibroma (MACF). As this category is new and rare now, we described here a case of MACF and reviewed systematically the literature for the appropriate treatment.

## Materials and methods

We treated a case of MACF, a new diagnostic category, with a long term history. And we searched previous cases using PubMed search system from 1966 to July 2015 with the terms “mitotically active cellular fibroma”, “ovary”, and “fibrosarcoma”. Then we analyzed and clarified that data of the cases have been recorded for finding important factors.

## Results

### Case description

**Fig. 1:**
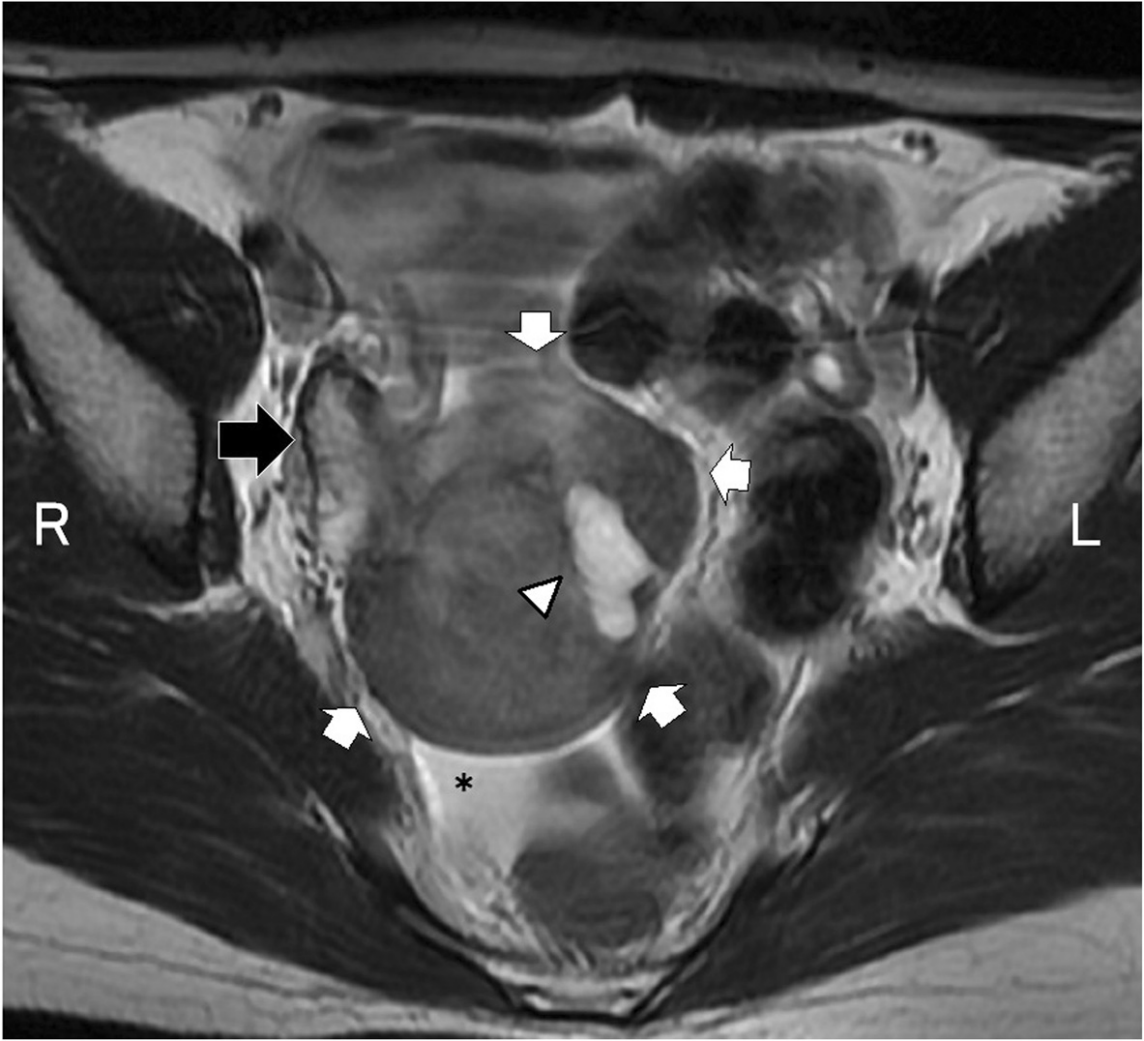
MRI appearance of the tumor. Axial T2-weighted MR image showed a well-circumscribed mass (white arrows) including cystic component (arrowhead) adjacent to the right ovary (black arrow) as having inhomogeneous low signal intensity. Small amount of ascites was noted (*)

**Fig. 2:**
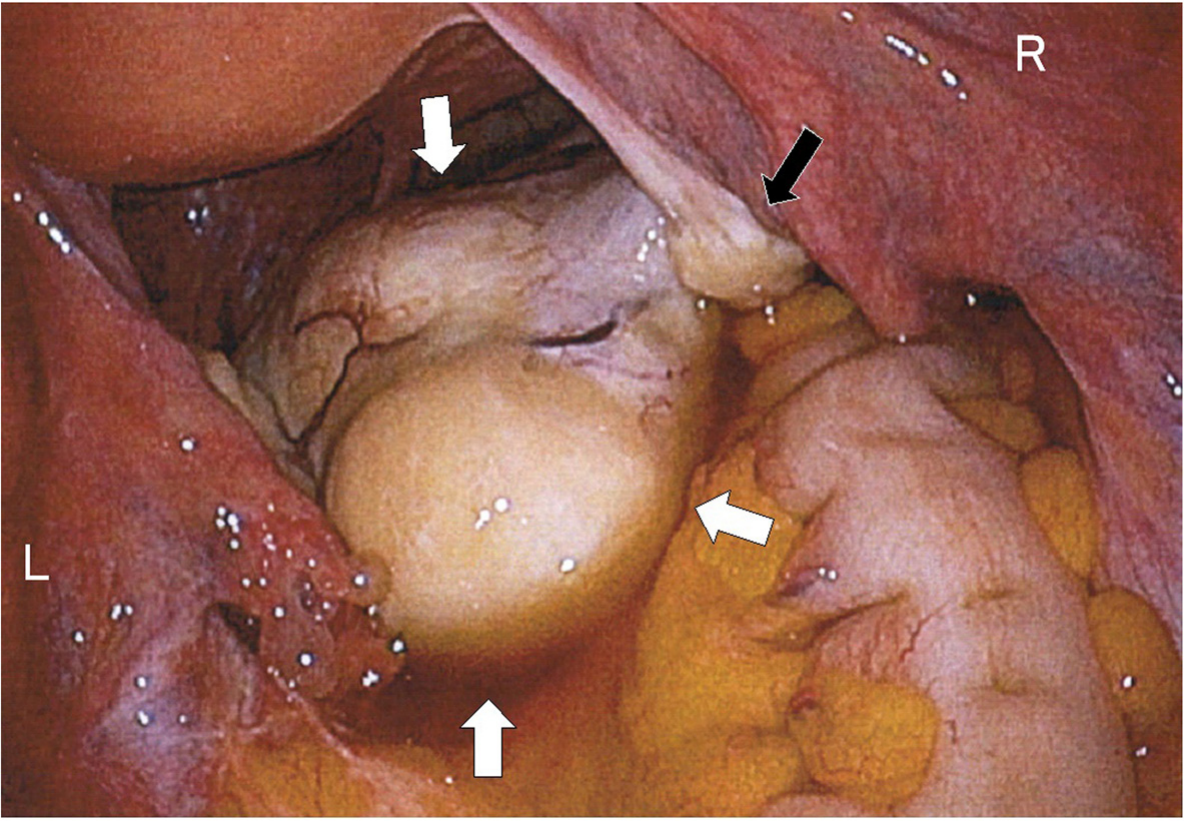
Laparoscopic findings of the tumor. The tumor (white arrows) in the Douglas pouch protruded from right ovary (black arrow) without adhesion

**Fig. 3:**
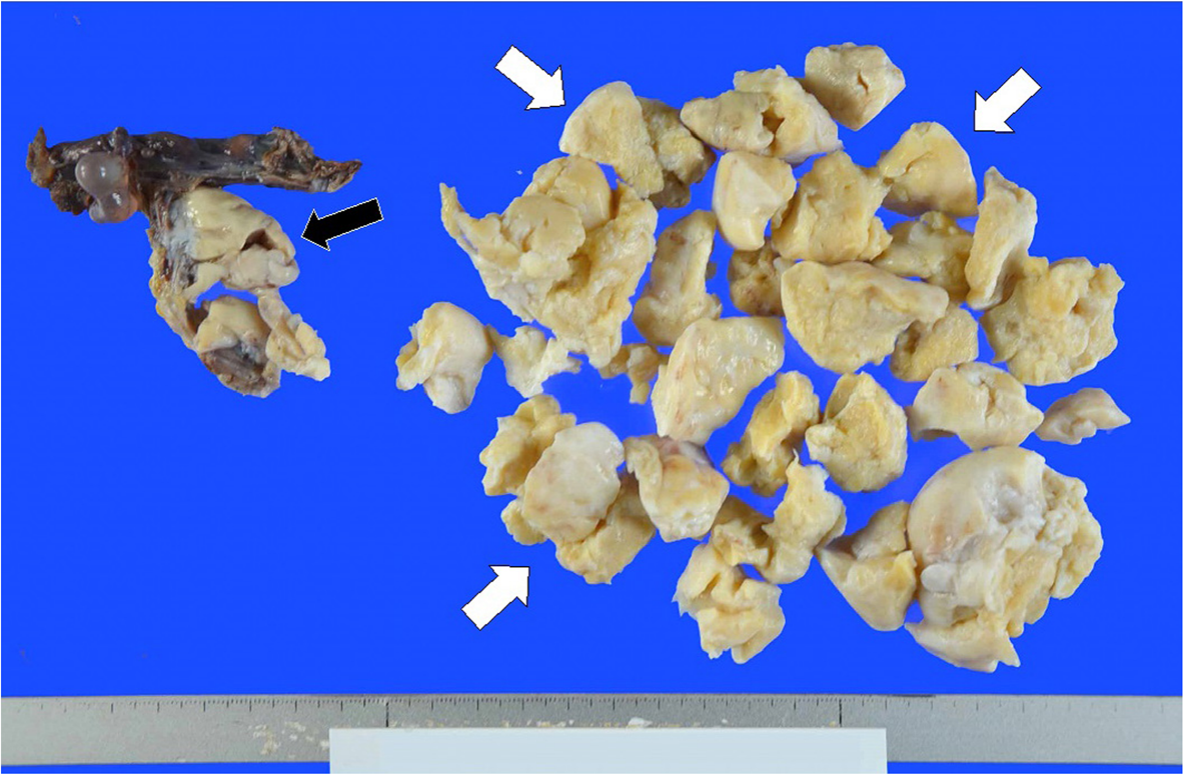
Macroscopic appearance of resected right oophorosalpinx. The sectioned surface of the tumor was solid and light-yellow without hemorrhagic necrosis. (right ovary: black arrow)

A 36-year-old woman (gravida 3, para 3) consulted a doctor for further examination of an ovarian tumor. She had been diagnosed with a right-sided 6-cm ovarian tumor 10 years previously, since her first pregnancy at another hospital. Pelvic examination showed an adnexal hard mass without tenderness on the right side. Vaginal echography and pelvic computed tomography (CT) showed a 6-cm ovarian solid tumor. Magnetic resonance imaging (MRI) revealed a 61-mm solid tumor with heterogeneous low signal intensity in the pelvis. As such, a benign tumor such as a fibroma/fibrothecoma or malignant tumor such as a germ cell tumor or lymphoma was suspected (Fig. 1). Serum levels of tumor markers were negative for carcinoembryonic antigen (CEA), CA125, CA19–9, squamous cell carcinoma (SCC), human chorionic gonadotropin (HCG) and alpha fetoprotein (AFP). Laparoscopic surgery was performed to confirm diagnosis and direct treatment after 8 months from the third delivery. Laparoscopic observation revealed that the tumor in the Douglas pouch protruded from the right ovary without adhesion (Fig. 2). Subsequently, right salpingo-oophorectomy was performed. The tumor was cut into small pieces in the bag, which was then extracted from the laparoscopic wound at the umbilicus without scattering in the abdominal cavity. The sectioned surface of the tumor was solid, light-yellow and without hemorrhagic necrosis (Fig. 3). Intraoperative cytology of ascitic fluid was negative, and intraoperative diagnosis of a frozen section revealed benign fibrous tumor as atypical cells were not observed.

**Fig. 4:**
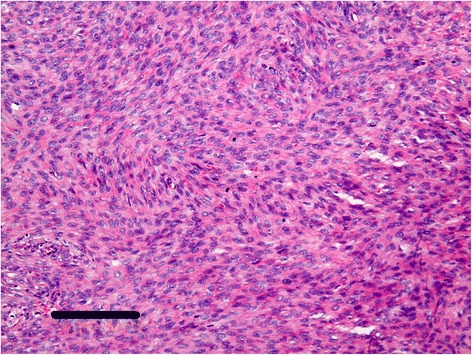
Macroscopic appearance of the tumor. The cells are spindle shaped and arranged in intersecting bandles. (bar = 200 μm)

**Fig. 5:**
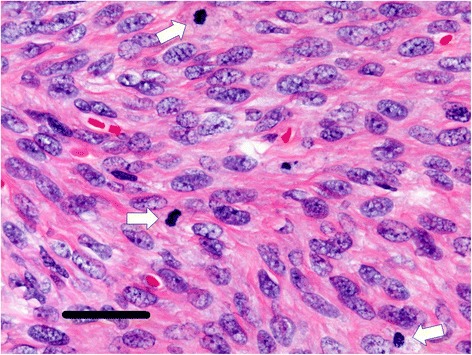
Macroscopic appearance of the tumor. The cells having spindled to ovoid shaped nuclei with moderate mitotic rate (mitoses: white arrows) but without significant nuclear atypia. (bar = 50 μm)

In paraffin sections, the tumor cells were spindle shaped and arranged in intersecting bundles (Fig. 4). They had ill-defined cytoplasmic borders and spindled to ovoid hyperchromatic nuclei with a moderate mitotic rate (10 per 10 HPF) but without significant nuclear atypia (Fig. 5). The cystic lesion was unclear. On immunohistochemistry, vimentin, progesterone receptor (PR), CD10, CD56 and Wilms tumor gene (WT-1) were positive, alpha-inhibin was focally positive, and pan cytokeratin (AE1/AE3), estrogen receptor (ER), calretinin and epithelial membrane antigen (EMA) were negative. Ki-67 index was 8.7 %. Pathologic findings and the case’s long-term clinical history led to the diagnosis of MACF of the ovary. She had a good clinical course after surgery for 6 months.

### Review the literature

In connection with ovarian tumor, five cases were found by a PubMed search system and shown in Table 1 [2–6]. Laparotomy was selected in all cases. Long-term local recurrence has also been seen in one case but all cases were alive at time of publication. However, there was no specific tumor marker or immunochemical staining.

**Table 1:** Cases of mitotically active cellular fibroma of the ovary

Author	Year	Case no.	Age (y)	Side	Size(cm)	Tumor marker	Mitoses (MF/10HPF)	Surgery	Additional therapy	Positive	Weak, focally	Negative	ki-67 index	Follow-up time (mo)	Status at last follow-up
Kaku et al. [2]	2007	1	32	Left	6.6 × 6,0 × 4.4	CA 125(-), CEA(-)	17	LSO	-	SMA, HHF35, vimentin, PR, ki-67	ER	desmin	ND	12	NED
Bucella et al. [3]	2009	2	65	ND	10(1st), 12 × 10 × 9 (2nd), 8 (3rd)	-	4	TAH, BSO, reduction twice	Tamoxifen	vimentin	α-inhibin, actin, CD99	desmin, h-caldesmon, CD10, HMB-45, c-kit	9	60 + 6 + 6	Recurrence twice, NED
Monterio et al. [4]	2012	3	13	Right	19 × 15 × 12	CA125(453), AFP(-), HCG(-), CEA(-), CA199(-), CA153(-)	5–7	RSO, OMT	-	ND	ND	ND	ND	36	NED
Wu et al. [5]	2014	4	76	Right	9 × 6 × 5	CA125(-), CA153(-), CA199 (-),AFP (-), CEA(-), SCC(-)	5–9	TAH, BSO	-	vimentin, α-inhibin, ER, PR	CD56, CD99	cytokeratin, EMA, CD10, HMB45, S-100, calretinin, CD34, CD117, Dog-1	10	ND	ND
Zong et al. [6]	2014	5	39	Left	10 × 7 × 4	CA125(41), HCG(-)	3–5	TAH, LSO, OMT, LD	-	CD99, CK, SMA, vimentin, ER, PR, S-100	-	CD10, CK7, EMA, desmin	10	66	NED
This case	2015	6	36	Right	6	CEA(-), CA125(-), CA199(-), SCC(-), HCG(-), AFP(-)	10	RSO	-	vimentin, PR, CD10, CD56, WT1	α-inhibin	AE1/AE3, ER, calretinin, EMA	8.7	6	NED

ND, not described; CEA, carcinoembryonic antigen; (-), within normal range; AFP, α-feto protein; HCG, human chorionic gonadotropin; SCC, squamous cell carcinoma; TAH, total abdominal hysterectomy; BSO, bilateral salpingo-oophorectomy; OMT, omentectomy; LD, lymphadenectomy; SMA, smooth muscle actin; HHF35, anti-muscle-specific actin (clone; HHF35); PR, progresterone receptor; ER, estrogen receptor; CK, cytokeratin; WT, Wilms tumor; HMB, human melanoma black; EMA, epithelial membrane antigen; NED, no evidence of the diseade

## Discussion

In the 2003 WHO histological classification, ovarian cellular fibrous tumors with mitotic figures 3 or less per 10 HPF and no severe nuclear atypia were defined as cellular fibroma, while the fibrous tumors with mitotic figures >4 per 10 HPF and severe nuclear atypia were defined as fibrosarcoma. However, ovarian tumors with mitotic figures >4 per 10 HPF but no severe nuclear atypia were not categorized. These kinds of tumors were mostly diagnosed as ovarian fibrosarcoma. However, the clinicopathologic characteristic of this tumor is that it is significantly different from the malignant behaviors of ovarian fibrosarcoma. In 2006, Irving et al. first defined these kind of ovarian tumors as ‘MACF’ [7]. In the 2014 WHO histological classification, a new definition was described: “mitotic activity of 4 per 10 HPFs in an ovarian cellular fibromatous neoplasm in the absence of moderate to severe atypia does not signify a fibrosarcoma. In such cases, a diagnosis of MACF is made.”

To date, only five cases of MACF of the ovary have been reported in the literature. All cases were alive at time of publication. However, there were no specific diagnostic or prognostic factors.

Our patient is the first case of MACF of the ovary treated with laparoscopic surgery. Ovarian benign fibrous tumors such as fibroma/fibrothecomas are often misdiagnosed as uterine myomas and malignant ovarian tumors preoperatively. However, laparoscopic surgery has been used for the management of ovarian fibroma/fibrothecomas [8, 9] and uterine myomas [10]. Histological diagnosis should be very important and include intraoperative consultation. Even if many mitoses are observed, it is imperative that we do not misdiagnose fibrosarcoma, in order to avoid excessive treatment. In our case, the tumor was benign both historically and histologically.

## Conclusions

We report a case of MACF with a long term history treated by laparoscopic surgery. MACF of the ovary is a newly defined category and few cases have been reported, while prognostic factors have also not yet been fully characterized. Treatment is still not well established therefore long-term clinical follow-up including image processing, such as echography, CT, MRI etc., is necessary.

### Consent

Written informed consent was obtained from the patient for publication of this Case report and any accompanying images. A copy of the written consent is available for review by the Editor-in-Chief of this journal.

## Abbreviations

HPF: high-power fields
WHO: World Health Organization
MACF: mitotically active cellular fibroma
CT: computed tomography
MRI: magnetic resonance imaging
CEA: carcinoembryonic antigen
SCC: squamous cell carcinoma
HCG: human chorionic gonadotropin
AFP: alpha fetoprotein
PR: progesterone receptor
WT-1: Wilms tumor gene
AE1/AE3: pan cytokeratin
ER: estrogen receptor
EMA: epithelial membrane antigen

## References

[1] Prat J, Scully RE (1981). Cellular fibromas and fibrosarcomas of the ovary: a comparative clinicopathologic analysis of seventeen cases. Cancer.

[2] Kaku S, Takeshima N, Akiyama F, Furuta R, Hirai Y, Takizawa K (2007). A unique fibrous tumor of the ovary: fibrosarcoma or mitotically active cellular fibroma?. Anticancer Res.

[3] Bucella D, Limbosch JF, Buxant F, Simon P, Fayt I, Anaf V, Noel JC (2009). Recurrence of mitotically active cellular fibroma of the ovary. Obstet Gynecol Int.

[4] Monteiro SB, Costa A, Paiva V (2012). Mitotically active cellular ovarian fibroma with Meigs’ syndrome and elevated CA-125: towards fertility preservation. J Pediatr Adolesc Gynecol.

[5] Wu H, Xie J, Huang W, Wu J (2014). Mitotically active cellular fibroma of the ovary: a case report and a review of the literature. Eur J Gynaecol Oncol.

[6] Zong L, Lin M, Fan X (2014). Mitotically active cellular fibroma of ovary should be differentiated from fibrosarcoma: a case report and review of literature. Int J Clin Exp Pathol.

[7] Irving JA, Alkushi A, Young RH, Clement PB (2006). Cellular fibromas of the ovary: a study of 75 cases including 40 mitotically active tumors emphasizing their distinction from fibrosarcoma. Am J Surg Pathol.

[8] Son CE, Choi JS, Lee JH, Jeon SW, Hong JH, Bae JW (2011). Laparoscopic surgical management and clinical characteristics of ovarian fibromas. Jsls.

[9] Cho YJ, Lee HS, Kim JM, Joo KY, Kim ML (2013). Clinical characteristics and surgical management options for ovarian fibroma/fibrothecoma: a study of 97 cases. Gynecol Obstet Invest.

[10] Dubuisso JB, Fauconnier A, Babaki-Fard K, Chapron C (2000). Laparoscopic myomectomy: a current view. Hum Reprod Update.

